# The impact of phospholipid transfer protein (PLTP) on lipoprotein metabolism

**DOI:** 10.1186/1743-7075-9-75

**Published:** 2012-08-16

**Authors:** Xian-Cheng Jiang, Weijun Jin, Mahmood M Hussain

**Affiliations:** 1 Department of Cell Biology, Downstate Medical Center, State University of New York, 450 Clarkson Ave., Box 5, Brooklyn, NY, 11203, USA

## Abstract

It has been reported that phospholipid transfer protein (PLTP) is an independent risk factor for human coronary artery disease. In mouse models, it has been demonstrated that PLTP overexpression induces atherosclerosis, while its deficiency reduces it. PLTP is considered a promising target for pharmacological intervention to treat atherosclerosis. However, we must still answer a number of questions before its pharmaceutical potential can be fully explored. In this review, we summarized the recent progresses made in the PLTP research field and focused on its effect on apoB-containing- triglyceride-rich particle and HDL metabolism.

## Phospholipid transfer protein (PLTP)

PLTP belongs to a family of lipid transfer/lipopolysaccharide-binding proteins, including cholesterol ester transfer protein (CETP), lipopolysaccharide-binding protein (LBP) and bactericidal/permeability increasing protein (BPI) [1]. It is a monomeric protein of 81 kDa [2]. Besides phospholipids, PLTP efficiently transfers diacylglycerol, α-tocopherol, cerebroside, and lipopolysaccharides [3]. Therefore, plasma PLTP is also a nonspecific lipid transfer protein. It has also been reported that there are two forms of lipoprotein-associated PLTP proteins. Active plasma PLTP is associated with apoA-I- containing lipoproteins (about 160 kDa in size) and inactive one is associated with apoE-containing lipoproteins (about 520 kDa in size) [4-6]. However, we still do not know why there are two forms of PLTP in the circulation?

PLTP is expressed ubiquitously [2,7]. The highest expression levels in human tissues were observed in ovary, thymus, placenta, and lung [2]. Taking into account the organ size involved, liver and small intestine appear to be important sites of PLTP expression. It was also shown that PLTP is highly expressed in macrophages [8-10] and in atherosclerotic lesions [11,12].

The liver is one of the major sites of lipoprotein production and degradation, as well as of PLTP expression. To address the impact of liver-expressed PLTP on lipoprotein metabolism, we created a mouse model that expresses PLTP in the liver acutely and specifically, with a PLTP-null background. We found liver expressed PLTP mice have about 25 % plasma PLTP activity compared to that of WT mice [13]. We also created liver-specific KO mice and found that the KO mice have 25 % less plasma PLTP activity than that of controls (Yazdanyar and Jiang, unpublished observation). These results indicated that liver-generated-PLTP makes about 25 % contributions to the PLTP activity in the circulation.

## PLTP regulation

PLTP activity and mRNA can be regulated by many factors. A high-fat high-cholesterol diet causes a significant increase in PLTP activity and in mRNA levels [7]. After lipopolysaccharide injection, plasma PLTP activity is significantly decreased, and this is associated with a similar decrease in PLTP mRNA levels in the liver and adipose tissues [7]. PLTP expression and activity can be upregulated by glucose [14] and down regulated by insulin [15,16]. It has been reported that diacylglyceride can also regulate PLTP activity [17].

PLTP promoter contains farnesoid X-activated receptor (FXR) and peroxisome proliferator-activated receptor (PPAR) binding motifs. The promoters of human and mouse PLTP genes show five consensus sequences for the transcription factors Sp1 and AP2 that are necessary for PLTP transcription [18,19]. The transcriptional activity of PLTP gene was significantly increased by chenodeoxycholic acid and fenofibrate, suggesting that FXR and PPAR are probably involved in the process [18]. We [8] and another group [20] independently showed that PLTP expression can also be upregulated by liver X receptor (LXR). The PLTP promoter contains a high-affinity LXR response element that is bound by LXR/RXR heterodimers in vitro, and is activated by LXR/RXR in transient-transfection studies [21]. A previous report indicated that LXR agonists activate triglyceride synthesis and PLTP transcription by activating SREBP-1c [22].

## PLTP and cholestery ester transfer protein (CETP)

Although PLTP and CETP show moderate homology of sequence [2] and similar structural features [1,23], they show no overlap in their in vivo functions. This was demonstrated in our study by preparing CETP transgenic/PLTP KO mice; the expression of CETP did not rescue the low HDL phenotype of PLTP deficiency. In fact the phenotypes were additive, resulting in markedly reduced HDL levels in the CETPTg/PLTP KO mouse [24]. However, there is an interaction between PLTP and CETP. It has been reported that purified PLTP enhances cholestery ester transfer from HDL_3_ to VLDL [25], even though PLTP has no such transfer activity of its own. Moreover, CETP transgenic/PLTP KO mice has significantly lower CETP activity than that of CETP transgenic mice [24].

## PLTP and HDL metabolism

Plasma PLTP mediates net transfer of phospholipids from apoB-containing-triglyceride-rich lipoprotein into HDL, and also exchanges phospholipids between lipoproteins [26,27]. Additionally, it has been shown that PLTP can act like a putative fusion factor to enlarge HDL particles [28]. Huuskonen et al. reported that phospholipid transfer activity is a prerequisite for efficient PLTP-mediated HDL enlargement [29]. Rye et al. reported that enrichment of triglyceride in the HDL core could promote such fusion [30].

Overexpression of PLTP in mice using adenovirus and adenovirus-associated virus resulted in a 10- to 40-fold increase in plasma PLTP activity [31,32]. These mice were characterized by increased preβ-HDL levels but decreased α-HDL cholesterol levels. PLTP expression mediated by adenovirus-associated virus (AAV) showed a prolonged pattern of overexpression that resulted in a significant decrease in total cholesterol and HDL cholesterol in C57BL/6 mice [32]. We prepared PLTP transgenic mice and found that the preβ HDL is significantly increased [33]. Transgenic mice that overexpress human PLTP at high levels were also generated. Compared with WT mice, they showed a 2.5- to 4.5-fold increase in PLTP activity in plasma. This resulted in a 30 to 40% reduction of plasma HDL cholesterol levels, but a 2- to 3-fold increase in the formation of PreB-HDL [34]. Overall, PLTP overexpression causes a significant reduction in plasma HDL levels but increases preβ-HDL.

So far, no PLTP deficiency has been found in humans. The most useful information about PLTP deficiency was obtained from PLTP gene knockout (KO) mice. These mice show a complete loss of phosphatidylcholine (PC), phosphatidylethanolamine, phosphatidylinositol, sphingomyelin but a partial loss of free cholesterol transfer activities [35]. Moreover, the in vivo transfer of ^3^ H] phosphatidylcholine from VLDL to HDL does not occur in PLTP KO mice. On a chow diet, these mice showed a marked decrease in HDL-PL, HDL-FC, and apoA-I, demonstrating the important role of PLTP-mediated transfer of surface components of triglyceride-rich lipoprotein in the maintenance of HDL levels [35]. Additionally, the HDL from the PLTP KO mice was enriched in protein but was deficient in PC. Turnover studies showed a 4-fold increase in the catabolism of HDL protein and cholesterol in PLTP KO mice compared with WT mice [36,37]. Overall, PLTP deficiency causes a significant reduction in plasma HDL cholesterol levels.

Recently, we compared HDL isolated from transgenic, wild type and knockout mice and found that: 1) HDLs isolated from different mice have different sizes, the order being as follows: PLTP transgenic > WT > PLTP KO; 2) the HDLs have different inflammatory index, the order being as follows: PLTP transgenic > WT > PLTP KO; and 3) the HDLs have different lipid compositions. The order of HDL- cholesterol levels is WT > PLTP transgenic > PLTP KO; the order of HDL total phospholipids is WT > PLTP Transgenic = PLTP; the order of triglyceride is WT > PLTP transgenic > PLTP KO (Yeang, Navab, and Jiang, unpublished observation). These studies indicate that PLTP might play an important role in determining plasma HDL size, inflammatory index and lipid composition. We also found that liver-specific PLTP deficiency significantly decreases HDL and apoA-I levels (Yazdanyar and Jiang, unpublished observation).

## PLTP in cholesterol efflux/reverse cholesterol transport

PLTP is highly expressed and regulated in macrophage cells and this suggests its potential involvement in lipid efflux. However, the role of PLTP in reverse cholesterol transport (RCT) (most of the studies were based on mouse macrophage cholesterol efflux model) is controversial. There are reports which indicate that PLTP might promote [38][39] or inhibit [40][41] or have no effect [8] on cell cholesterol efflux. Differences in various published reports might be because these studies did not compare same amounts of HDL.

Oram et al. reported that exogenous PLTP can promote HDL-mediated cholesterol efflux through ABCA1 pathway [38]. We also found that recombinant PLTP (50 ng/ml) together with 0.8 nmole/ml HDL promotes HDL mediated cholesterol efflux (Yeang and Jiang, unpublished observation). PLTP appears to function as an intermediary in the transfer of excess cellular lipids to lipoproteins through its interaction with ABCA1 [38]. It was also indicated that an amphipathic helical region of the N-terminal barrel of PLTP is critical for ABCA1-dependent cholesterol efflux [39]. Furthermore, Lee-Rueckert et al. studied the ABCA1-dependent efflux of cholesterol from peritoneal macrophages derived from PLTP-deficient mice and compared it with cholesterol efflux from wild-type macrophages. They found that cholesterol efflux from PLTP-deficient macrophage foam cells is defective and that the defect can be corrected by robust stimulation of the ABCA1-dependent pathway. These results support an intracellular role for endogenous macrophage PLTP in ABCA1-mediated cholesterol efflux from macrophage foam cells [10]. As mentioned previously, PLTP is present in plasma as two forms, a highly active (HA-PLTP) and a lowly active (LA-PLTP) form [4,21]. Vikstedt et al. reported that incubation of HDL in the presence of HA-PLTP resulted in the formation of preβ-HDL and caused a 42% increase in macrophage cholesterol, while LA-PLTP neither formed preβ-HDL nor increased cholesterol efflux. However, neither HA- nor LA-PLTP enhanced cholesterol efflux to lipid-free apoA-I [42]. Based on the above results, PLTP may promote macrophage cholesterol efflux.

On the other hand, Moerland et al. reported that in cholesterol efflux studies from macrophages, HDL isolated from human PLTP/ human apoA-I double transgenic mice was less efficient than HDL isolated from human apoA-I transgenic mice[40]. Furthermore, it was found that the largest subfraction of the HDL particles present in the double transgenic mice was markedly inferior as a cholesterol acceptor, as no labeled cholesterol was transferred to this fraction. These data demonstrate that the action of human PLTP in the presence of human apoA-I results in the formation of a dysfunctional HDL subfraction, which is less efficient in the uptake of cholesterol from cholesterol-laden macrophages [43]. The same group of researchers investigated the role of systemic and peripheral PLTP in macrophage cholesterol efflux and reverse cholesterol transport in vivo. They found that macrophage cholesterol efflux and reverse cholesterol transport to feces is impaired in PLTP transgenic mice, and that elevation of macrophage-PLTP does not affect reverse cholesterol transport, indicating that higher systemic PLTP levels may promote atherosclerosis development by decreasing the rate of reverse cholesterol transport [41]. The same experiment needs to be performed in PLTP deficient mice. Based on the above results, PLTP may inhibit macrophage cholesterol efflux.

Contradictory results are also observed in human studies. De Vries et al. reported that cholesterol efflux from fibroblasts to the HDL from normotriglyceridemic diabetic plasma is unchanged, while efflux to HDL by the source of hypertriglyceridemic diabetic plasma is enhanced, with concomitant increased plasma PLTP activity [44]. However, Attia et al. indicated that in diabetic patients with or without CHD, PLTP activity was consistently increased in comparison with the control group [45].

Apolipoprotein F (ApoF) is known as lipid transfer inhibitor protein (LTIP) based on its ability to inhibit lipid transfer between lipoproteins ex vivo. ApoF overexpression reduces HDL cholesterol levels in mice by increasing clearance of HDL-CE [46], however, whether PLTP is involved in this process is still unknown.

## PLTP and apoB-containing lipoprotein (BLp) metabolism

ApoB is the major protein component of VLDL and chylomicrons (CM), which transport triglyceride from the liver and intestine, respectively, into the bloodstream [47]. ApoB exists in two forms, apoB48 and apoB100 [48,49]. Increased hepatic BLp synthesis is the principal defect in subjects with familial combined hyperlipidemia [50,51], and is also an important component of the dyslipidemia of diabetes and obesity [52,53]. Accumulating evidence suggests that the formation of apoB100-BLp [54] and apoB48-BLp [55,56] is accomplished sequentially. The "two-step" model postulates that the initial product is a primordial particle, formed during apoB translation in the endoplasmic reticulum (ER). It is clear that MTP is involved in the early stage (1st step) of apoB lipidation. However, the mechanism involved in the later stage (2nd step) in which the apoB-containing primordial particle fuses with apoB-free/triglyceride-rich lipid droplets is still not well understood [57]. Abundant triglyceride availability is essential, but it alone is not sufficient to drive BLp assembly. This is exemplified by studies using hepatic cells treated with n-3 fatty acids [58,59] or insulin [60], in which active triglyceride synthesis does not result in VLDL production. In certain hepatoma cell lines (e.g., HepG2 cells), triglyceride synthesis can be effectively stimulated by oleate, but formation of VLDL is not achieved [61].

We unexpectedly found that PLTP deficiency causes a significant impairment in hepatic secretion of VLDL [62]. Likewise, it has been reported that animals overexpressing PLTP exhibit hepatic VLDL over-production [63]. Associations of plasma PLTP activity with elevated apoB levels have been found in humans as well [64]. In a recent study, Dr. Lagrost’s group found that human PLTP transgenic rabbits showed a significant increase of BLp but not of HDL cholesterol in the circulation [65]. This might reflect the real situation in humans, since rabbits, like humans, are LDL mammals. Nevertheless, the surprising finding that PLTP affects BLp secretion from the liver has remained unexplained.

The contribution of hepatic synthesis of PLTP on plasma apoB-lipoproteins was investigated in several murine models that specifically expresses PLTP in the liver on a PLTP-null background, hepatic overexpression of PLTP was responsible for increased plasma PLTP activity, and increased VLDL production and circulating concentrations of apoB-containing lipoproteins, but had marginal effect on HDL and apoA-I levels [13]. Recently, we also found that liver-specific PLTP KO mice secrete significantly less apoB-containing particles from the liver compared with controls (Yazdanyar and Jiang, unpublished observation).

We have found that mouse small intestine expresses PLTP (Jiang XC, unpublished observation). We have also found a significant reduction in BLp-cholesterol secretion from enterocytes obtained from PLTP KO mice, compared with controls [66]. There are similarities between VLDL and chylomicron production in the liver and small intestine, respectively [57]**.** We believe that PLTP activity is involved in promoting 2nd step of BLp lipidation, since PLTP activity and triglyceride enrichment are two factors for PLTP-mediated HDL enlargement [29,30], a process similar to the 2nd step of BLp lipidation [57]. We proposed a model for this. We hypothesize that although PLTP has no triglyceride transfer activity, PLTP-mediated phospholipid transfer or exchange on the surface of primordial BLp and apoB-free/TG-rich lipid droplets would fuse two particles.

PLTP has vitamin E transfer activity that is important to maintain tissue and plasma vitamin E levels. It is known that vitamin E-enriched LDL from PLTP deficient mice is resistant to oxidation and also is much less active to induce monocyte chemotactic activity [37,67]. Over expression of PLTP decreases vitamin E content in LDL and increases its oxidation [32]. Therefore, PLTP deposits vitamin E from plasma to cells. Accumulating data suggest that the function of PLTP in tissues is different from its role in plasma. Studies on macrophage-derived PLTP has demonstrated that PLTP deficient macrophages have more basal cholesterol level and accumulate more cholesterol in the presence of LDL [68]. Supplementation of vitamin E in these animals normalizes the cholesterol phenotype [68]. We have shown that PLTP deficient hepatocytes secrete less apoB-containing lipoproteins and this is related to premature degradation caused by lacking vitamin E and increasing oxidation stress [69]. Hence, a major effect of PLTP on cellular physiology might be due to changes in cellular vitamin E levels and oxidative stress.

Overproduction of VLDL may be beneficial for preventing nonalcoholic fatty liver disease (NAFLD). However, plasma PLTP activity is positively associated with serum alanine aminotransferase and aspartate aminotransferase, two enzymes considered as predicts for NAFLD, in diabetes patients, and it has been suggested that PLTP may be a marker for NAFLD[70]. More importantly, PLTP deficiency does not cause lipid accumulation in the liver [62].

## PLTP in the innate immune system

Lipopolysaccharides (LPS) are amphipathic molecules that are localized in the outer leaflet of the outer membranes of gram-negative bacteria. They activate the innate immune system through a complex process involving Toll-like receptors (TLRs) and the MD-2, CD14, and lipopolysaccharide-binding protein (LBP) accessory proteins [71]. PLTP can transfer and neutralize LPS [72]. Based on PLTP KO mouse study, it has been shown that PLTP plays a physiologically relevant role in the disaggregation, binding, and transfer of LPS to lipoproteins [73]. Recently, it has been further shown that PLTP is essential in mediating the association of triacyl lipid A of LPS with lipoproteins, leading to extension of its residence time and to magnification of its proinflammatory and anticancer properties [72].

## PLTP and atherosclerosis

Genome-wide association studies (GWAS) have made spectacular advances in identifying genes associated with dyslipidemia and coronary heart disease (CHD) [74-77]. However, GWAS reports on PLTP are contradictory. It has been reported that two PLTP single-nucleotide polymorphisms (SNPs) are associated with lower PLTP activity, higher HDL levels, and a decreased risk of CHD [78]. On the other hand, SNPs near the PLTP gene are associated with higher PLTP activity, higher HDL, and lower TG levels [79].

PLTP expression is increased in different pathologies associated with increasing risk of CHD, such as obesity, insulin resistance, and types I and II diabetes [80]. We have found that serum PLTP activity is increased in CHD patients [81]. Moreover, PLTP activity is positively correlated with heart failure due to coronary artery ischemia [82] and low HDL levels [83]. Contradictorily, one study has shown that low PLTP is a risk factor for peripheral atherosclerosis [84]. It has been reported that immunoreactive PLTP was discovered in histological sections of human carotid artery [11,12]. It was colocalized with CD-68 positive macrophages, suggesting its production in situ. Synthesis of PLTP was further demonstrated in cultured macrophages and its expression was upregulated by acetylated LDL treatment [10,20]. Moreover, in the atherosclerotic segments, PLTP accumulated in extracellular matrixes, colocalizing with apoA-I, apoE, and biglycan [12].

In mouse models, it has been demonstrated that PLTP overexpression induces atherosclerosis [32,85], while its deficiency shows the opposite effect [62]. Two bone marrow transplantation studies indicated that PLTP expression by bone marrow derived cells increases LDL receptor KO mouse atherosclerotic lesion size [86,87]. However, other bone marrow transplantation studies indicated that local PLTP expression in macrophages could be protective as long as systemic PLTP levels are not markedly elevated [86,88,89]. In rabbits, PLTP overexpression increases atherosclerotic lesions after a high-fat diet feeding, compared with controls [65]. In general, elevation of systemic PLTP is a risk factor for atherosclerosis in animal models. Therefore, reductions in plasma PLTP might be beneficial.

## Conclusion

Plasma PLTP activity influences apoB-containing lipoprotein and HDL metabolism. Cellular PLTP activity influences apoB-containing lipoprotein production. PLTP activity may or may not have an effect on reverse cholesterol transport. Importantly, PLTP clearly has a notable role in the development of atherosclerosis. However, our knowledge about PLTP activity, especially inside the cells, is very limited. To better understand the role of PLTP in atherogenecity, we still need to explore PLTP-mediated lipoprotein metabolism. Further, more epidemiological studies are needed to gain insights into the role of PLTP in atherosclerosis. Lastly, discovery of humans with genetic PLTP deficiency would be a major step toward the elucidation of the role of this transfer protein in human lipoprotein metabolism and atherosclerosis.

## Abbreviation

PLTP: Phospholipid transfer protein; BLp: apoB-containing-triglyceride-rich particles; KO: gene knockout; Apo: Apolipoprotein; HDL: High density lipoprotein; VLDL: Very low density lipoprotein.

## Competing interests

The authors declare that they have no competing interests.

## Authors’ contributions

X-CJ wrote the review. WJ made corrections. MMH made corrections. All authors read and approved the final manuscript.
